# Christmas at the Hospitals

**Published:** 1888-01-07

**Authors:** 


### CHRISTMAS AT THE HOSPITALS.

### PAST AND PRESENT.

OUR forefathers would have thought that Christmas spent in the hospital was equivalent to penal discipline or exile to Siberia. When all the world outside was anticipating in a hundred different ways the joys of the season, the bare prospect of being shut off from friends and relations in a building devoted to the sick and dying must have been lugubrious in the extreme. Even the promise of the perennial roast beef and the extra allowance of beer or tea which a later generation has introduced into the Christmas fare of workhouses and many hospitals, would have little attraction to the patient who had friends at home whose society alone would compensate the most meagre fare. All these things now happily belong to the past. It was left to the present generation to alter them, and to render the great festival of the year as cheerful to the inmate of the hospital as to his sisters and brothers outside. The evergreen holly with its gladsome associations, the flowers which bloom in mid-winter, and the music which lends a charm to everything sublunary, are all brought into requisition to remind the patients that although they have parted from the busy world for a season, they are not forgotten by their friends, nor are their attendants insensible to their wants and desires. It would be almost an impossible task to give in detail the various plans and devices now so universally employed to amuse and interest the sick inmates of hospitals at this particular season, and it would appear to many a little bit incongruous to the *genius loci* that such things are even permitted. We will not attempt to combat "old fogeydom," but we feel sure that in no well-regulated establishment would anything be allowed that would prove prejudicial to the recovery of the patients, or injurious to the dying. In some hospitals it is customary to have concerts and dramatic performances either in the wards or in large rooms adjacent, where the patients can be conveniently carried, while the performances are rendered pleasanter by appropriate decorations, conceived in the best taste, and lighted up with Japanese lanterns and other fancy illuminations. Others, perhaps the larger number, affect a preference for holly wreaths and banks of winter flowers embedded in moss filling up the window-sill spaces. Christmas trees are seldom wanting in children's wards, and those are the best which provipe clothing for the little ones as well as toys and fruit. Here a gigantic snowball may be seen, containing no end of the same useful articles, and a live Father Christmas distributing them right and lft to the most needy; there a magic lantern, with its miraculous phantasmagoria, eliciting repeated applause, if we take our cue from the expression on the pale faces of the children, for they have scarcely strength enough to clap their hands. The presiding genius over all is a loving sister or nurse, whose mission in life she feels would be only half performed if she did not succeed in making everybody happy. There is no place like a hospital for raising the dead level of humanity; the one touch of nature which makes us all one family can nowhere be better realised and depicted than in hospitals at Christmas time.

### GUY'S.

On Boxing Day the patients at Guy's had their usual Christmas fare, comprising the time-honoured roast beef and plum pudding, supplemented by gifts of clothing, toys for the children, and other accessories liberally provided for by the students and other friends. All the wards were prettily decorated with holly and evergreens and Christmas flowers; and in the wards for women and children, Christmas trees laden with all the good things of the season were noticed to occupy a commanding position. During the afternoon a troupe of "nigger" minstrels, composed of students in costume, visited the several wards and gave a grotesque entertainment of singing and promiscuous buffoonery, which was greatly relished by the patients and their attendants. In the course of the evening the wards were illuminated by many hundreds of chinese lanterns gracefully arranged, which with other attractions gave a kind of scenic representation of the wonders of Aladdin's palace, tenanted by the lame, the halt, and the blind. Some vocal and instrumental music of a quieter and more refined kind wound up the entertainments, and old and young expressed themselves as all the better for the indulgence.

### UNIVERSITY COLLEGE.

THE Christmas festivities for the amusement of the patients at this hospital took place on Thursday, the 29th ult. The wards were decorated in the usual fashion with evergreens and appropriate mottoes, while in the centre of them stood Christmas trees or some substitute—the home of Father Christmas, a cave hewn out of solid rocks (of apples, oranges, and crackers), a snow-covered landscape, or a cottage hospital. Around these were grouped the presents, toys for the children, but more practical things—long desired articles of clothing—for the adults. One old woman declared, as she looked at a goodly parcel of flannel garments, that such a gift was worth five pounds to her. For the amusement of those patients who were able to leave the wards two concerts were given. At the first Mr. George Giddens, the popular comedian, gave a comic sketch, and if his efforts meet always with as much success as they did of Thursday evening he is greatly to be congratulated. One patient declared he had never laughed so much in his life; he feared that it had brought back the bronchitis that had lately left him, but considered the pleasure worth the risk. Later in the evening another concert, in which a number of ladies and gentlemen took part, was given with equal success. The happy looks of the patients, who thoroughly appreciated all the efforts made to entertain them, must have been sufficient reward to the sisters, the nurses, and the indefatigable secretary, Mr. Newton H. Nixon, for the trouble they had taken to give some reflection of Christmas joy to those under their charge.

### CENTRAL LONDON THROAT AND EAR HOSPITAL, GRAY'S INN ROAD.

THE patients in this Hospital had a Christmas treat on Thursday evening. Much of the credit of the evening's entertainment is due to the matron, Miss Danter, who devoted all her energy to the amusement of those under her care, and brought together several of her friends, who added much to the success and enjoyment of the evening. The wards were tastefully decorated with flags, banners, wreaths, Chinese lanterns, etc. After tea had been served, a very excellent concert was given by various ladies and gentlemen interested in the welfare of the Hospital.

### THE SOUTH INFIRMARY, CORK.

ON St. Stephen's night the operating theatre of the Cork South Infirmary and County Hospital presented a most edifying and encouraging spectacle, the spacious hall being tastefully decorated with evergreens, Chinese lanterns, and bunting. In the theatre it was pleasing to remark the genuine pleasure displayed on the countenances of the poor fellows whose beds were placed here. Patients suffering from broken limbs, from diseases of the spine, paralysis, and ailments which condemn them to a recumbent position, were thus enabled to enjoy the proceedings of the evening. Those benches were thronged with a motley array of nurses, patients, and friends, who, by their frequent applause, showed themselves pleased with the concert which took place. In the centre portion of the theatre were placed a number cf chairs for the ladies and gentlemen who took an active part in the proceedings. The third song was given by Dr. Tanner, M.P., in his usual vigorous style, and was encored. A vote of thanks was proposed by Dr. Tanner to Dr. Guisani, who is about to leave the institution, and whose past career was but indicative of the success which must attend the efforts of so capable and finished a gentleman. He praised the trustees, and especially Mr. S. H. Newsom, and spoke of the services rendered to the institution by an old and esteemed friend, with whom he had been acquainted since his childhood, the good and worthy matron, Miss Franklin. Dr. Guisani responded.

[*Other communications regarding Christmas Festivities unavoidably held over for want of space.*]

